# LncRNA‐mRNA expression profiles and functional networks in osteoclast differentiation

**DOI:** 10.1111/jcmm.15560

**Published:** 2020-07-26

**Authors:** Wenjie Liu, Zhaofeng Li, Zhaopeng Cai, Zhongyu Xie, Jinteng Li, Ming Li, Shuizhong Cen, Su'an Tang, Guan Zheng, Guiwen Ye, Hongjun Su, Shan Wang, Peng Wang, Huiyong Shen, Yanfeng Wu

**Affiliations:** ^1^ Department of Orthopedics The Eighth Affiliated Hospital Sun Yat‐sen University Shenzhen China; ^2^ Department of Orthopedics Sun Yat‐sen Memorial Hospital Sun Yat‐sen University Guangzhou China; ^3^ Department of Orthopedics Nanfang Hospital Southern Medical University Guangzhou China; ^4^ Center for Biotherapy Sun Yat‐sen Memorial Hospital Sun Yat‐sen University Guangzhou China

**Keywords:** ceRNA, expression profile, lncRNA, osteoclast differentiation

## Abstract

Human osteoclasts are differentiated from CD14^+^ monocytes and are responsible for bone resorption. Long non‐coding RNAs (lncRNAs) have been proved to be significantly involved in multiple biologic processes, especially in cell differentiation. However, the effect of lncRNAs in osteoclast differentiation is less appreciated. In our study, RNA sequencing (RNA‐seq) was used to identify the expression profiles of lncRNAs and mRNAs in osteoclast differentiation. The results demonstrated that expressions of 1117 lncRNAs and 296 mRNAs were significantly altered after osteoclast differentiation. qRT‐PCR assays were performed to confirm the expression profiles, and the results were almost consistent with the RNA‐seq data. GO and KEGG analyses were used to predict the functions of these differentially expressed mRNA and lncRNAs. The Path‐net analysis demonstrated that MAPK pathway, PI3K‐AKT pathway and NF‐kappa B pathway played important roles in osteoclast differentiation. Co‐expression networks and competing endogenous RNA networks indicated that ENSG00000257764.2‐miR‐106a‐5p‐TIMP2 may play a central role in osteoclast differentiation. Our study provides a foundation to further understand the role and underlying mechanism of lncRNAs in osteoclast differentiation, in which many of them could be potential targets for bone metabolic disease.

## 1 INTRODUCTION

Human osteoclasts are differentiated from CD14^+^ monocytes under the induction of macrophage colony stimulating factor (M‐CSF) and receptor activator of NF‐κB ligand (RANKL).^1^, ^2^ Tartrate‐resistant acid phosphatase (TRAP) and cathepsin K (CTSK) are the osteoclast differentiation markers and are in charge of bone resorption.^3^ During osteoclast differentiation, mononuclear cells fuse together and transform into multinucleated osteoclasts.^4^, ^5^ The actin cytoskeleton of osteoclasts is rearranged and a tight junction between the bone surface and basal membrane arises to form a sealing zone.^3^, ^6^ This sealing zone is acidified by hydrogen ions, and then, TRAP and CTSK are secreted to resorb bone and an absorption pit is formed.^3^, ^7^ In normal conditions, osteoclasts and osteoblasts maintain the balance of bone metabolism.^8^ Unfortunately, abnormal differentiation of osteoclasts occurs in many skeletal diseases such as giant cell tumours of the bone, multiple myeloma, osteoporosis and inflammatory rheumatic diseases.^9^, ^10^, ^11^, ^12^ Over the past decades, increasing numbers of regulating factors involved in the differentiation of osteoclasts have been discovered, including cytokines, molecules of signalling pathways, natural compounds and others.^13^, ^14^ However, the epigenetic regulation of osteoclast differentiation that leads to the dysregulation of osteoclasts is less well known.

Epigenetics analyse the heritable changes in gene expression when the nucleotide sequence of a gene does not change.^15^, ^16^ Long non‐coding RNAs (lncRNAs) belong to a kind of epigenetic regulation. Transcripts of lncRNAs are usually >200 nt and they do not encode proteins.^17^, ^18^, ^19^ Much of the non‐coding genome has historically been regarded as junk DNA.^20^ However, with advances in sequencing technology, an in‐depth examination of the non‐coding genome has been achieved; this revealed that the rate of non‐coding genome is more than 98%.^20^ LncRNAs are now known to participate in many biological processes, such as cell proliferation, differentiation and apoptosis.^21^, ^22^, ^23^, ^24^ However, little is known about the roles of lncRNAs playing in osteoclast differentiation.

LncRNAs carry out their functions through diverse mechanisms involving miRNAs, mRNAs and proteins.^25^, ^26^ miRNAs have emerged as important regulators of osteoclast differentiation.^27^, ^28^ Recent studies demonstrate that lncRNAs serve as competing endogenous RNAs (ceRNAs) by competing for binding to miRNAs.^29^, ^30^, ^31^ Studying this novel RNA crosstalk will provide significant insight into gene regulatory networks of osteoclast differentiation.

In this study, we explored the lncRNA‐mRNA expression profiles in human osteoclast differentiation by RNA‐seq and confirmed the expression profiles by qRT‐PCR assays. Moreover, GO and KEGG analyses were used to predict potential cellular functions of these differentially expressed mRNA and lncRNAs. The Path‐net analysis, co‐expression networks and ceRNA networks were applied to predict gene regulatory networks of osteoclast differentiation. Our study provides a new perspective on the regulation of osteoclast differentiation by lncRNAs.

## 2 MATERIALS AND METHODS

### 2.1 Cell isolation

Peripheral blood was acquired from healthy donors with the approval of the Ethics Committee of Sun Yat‐sen Memorial Hospital. Peripheral blood mononuclear cells (PBMCs) were isolated using density gradient centrifugation with Ficoll‐Hypaque solution (GE Healthcare Life Sciences, Pittsburgh, USA). Then, CD14^+^ monocytes were isolated with magnetic beads according to protocol (Miltenyi Biotech, Bergis‐Gladbach, Germany).

### 2.2 Osteoclast differentiation

To induce osteoclast differentiation, CD14^+^ monocytes (2.5 × 10^5^/cm^2^) were cultured in α‐minimum essential medium (α‐MEM) containing 10% FBS supplemented with 25 ng/mL M‐CSF (Peprotech) and 50 ng/mL RANKL (Peprotech, Rocky Hill, NJ, USA). All the cells were cultured in 5% CO_2_ at 37°C and the culture medium was changed every 3 days.

### 2.3 Tartrate‐resistant acid phosphatase staining

CD14^+^ monocytes (2.5 × 10^5^/cm^2^) were plated onto 24‐plate wells and cultured with 25 ng/mL M‐CSF and 50 ng/mL RANKL to induce osteoclasts. On day 9, cells were stained for tartrate‐resistant acid phosphatase (TRAP) activity with the leucocyte acid phosphatase kit (Sigma, St. Louis, MO, USA) according to the manufacturer's protocol. TRAP‐positive cells which had at least three nuclei were considered osteoclasts.

### 2.4 F‐actin assay

To detect podosomes in osteoclasts, we carry out F‐actin assays. After fixing with 4% paraformaldehyde for 5 minutes, cells were washed three times with PBS. Then, cells were stained with FITC‐conjugated phalloidin (Sigma) and 4′,6‐diamidino‐2‐phenylindole (DAPI) for 40 minutes. In the end, cells were washed with PBS thoroughly and observed with an Axio Observer fluorescence microscope (Carl Zeiss, Jena, Germany).

### 2.5 Bone resorption assay

CD14^+^ monocytes (2.5 × 10^5^/cm^2^) were plated onto bovine cortical slices and cultured with 25 ng/mL M‐CSF and 50 ng/mL RANKL for 15 days. Afterwards, the slices were fixed with 2.5% glutaraldehyde for 15 minutes and washed with PBS thoroughly. Subsequently, adherent cells on the slices were removed by ultrasonication in 0.5 mol/L NH_4_OH. Finally, the slices were gradient‐dehydrated in 70%, 95%, 100% ethanol, respectively, and stained with 1% (w/v) toluidine blue for 5 minutes.

### 2.6 Real‐time quantitative reverse transcription–polymerase chain reaction (qRT‐PCR)

Cells were treated with TRIzol (Invitrogen, Carlsbad, CA, USA) and total RNA was extracted. Then, 1 µg of RNA was transcribed to cDNA using a PrimeScript RT reagent kit (TaKaRa, Dalian, China) according to the manufacturer's protocol. The qRT‐PCR assay was carried out on a LightCycler^®^480 PCR system (Roche, Basel, Switzerland) using SYBR Premix Ex Taq (TaKaRa). The procedure for the quantitative‐PCR assay was 95°C for 30 seconds, 40 cycles at 95°C for 5 seconds and 60°C for 20 seconds. Each sample was divided into triplicate and the mean expression of each gene was calculated using the 2^−ΔΔCt^ method. Primers for all the genes are available in Table S1.

### 2.7 Western Blot analysis

After lysing in RIPA buffer (Sigma), cells were centrifuged at 10 000 g for 30 minutes. Then, proteins in the supernatant were quantified with a BCA assay kit (Sigma), separated by 10% sodium dodecyl sulphate‐polyacrylamide gel electrophoresis and electrotransferred onto polyvinylidene fluoride (PVDF) membranes (EMD Millipore Billerica, MA, USA). Subsequently, membranes were blocked for 60 minutes with 5% skim milk in TBST and incubated overnight with primary antibodies against Tubulin, TRAP or CTSK (each diluted 1:1000; Cell Signaling Technology, MA, USA) at 4°C. Afterwards, the membranes were washed with TBST and incubated for 60 minutes with appropriate horseradish peroxidase (HRP)–conjugated secondary antibodies (diluted 1:3000; Santa Cruz Biotechnology, CA, USA). The signal was detected using the Immobilon Western Chemiluminescent HRP Substrate (Millipore).

### 2.8 RNA‐seq analysis

Total RNA was extracted from cells after adding TRIzol (Invitrogen), and ribosomal RNA was removed using the Ribo‐Zero™ kit (Epicentre, Madison, WI, USA). According to the protocol of NEBNext^®^Ultra™ RNA Library Prep Kit for Illumina (NEB, Beverly, MA, USA), fragmented RNA was subjected to first strand and second strand cDNA synthesis followed by adaptor ligation and enrichment with a low‐cycle. The purified library products were evaluated with the Agilent 2200 TapeStation and Qubit^®^2.0 (Life Technologies, Gaithersburg, MD, USA). The libraries were paired‐end sequenced with the IlluminaHiSeq 3000 platform at Guangzhou RiboBio Co., Ltd. (Guangzhou, China).

### 2.9 GO and KEGG pathway analyses

GO analysis was performed using KOBAS3.0 software (available online: http://kobas.cbi.pku.edu.cn), including three domains: cellular component (CC), molecular function (MF) and biological process (BP). GO provides label classification of gene function and gene product attributes (http://www.geneontology.org). KEGG pathway analysis identified the significant enrichment of different pathways with KOBAS3.0 software (http://www.genome.jp/kegg).

### 2.10 Co‐expression network construction

The co‐expression network was established by calculating the Pearson correlation coefficient and *P*‐value between multiple genes. In this study, the transcripts were filtered using a COR of >0.85 and a *P*‐value of <0.05. The co‐expression network was illustrated using Cytoscape software (available online: https://cytoscape.org).

### 2.11 Competing endogenous RNA network construction

The lncRNAs and mRNAs were selected to predict miRNA targets using the miRbase. Then the miRNAs obtained from the predictions were screened with the miRanda and TargetScan programs. Afterwards, lncRNAs and mRNAs possessing miRNA recognition elements (MREs) for the targeted miRNAs were predicted using RNA22. The competitive endogenous RNA (ceRNA) network was established and illustrated using Cytoscape software.

### 2.12 Statistical analysis

Statistical analysis was conducted with SPSS 22.0 software (Chicago, IL, USA). Student's t test was used for the comparisons between two groups. A *P*‐value of <0.05 was considered significant.

## 3 RESULTS

### 3.1 Osteoclast differentiation and identification

**FIGURE 1:**
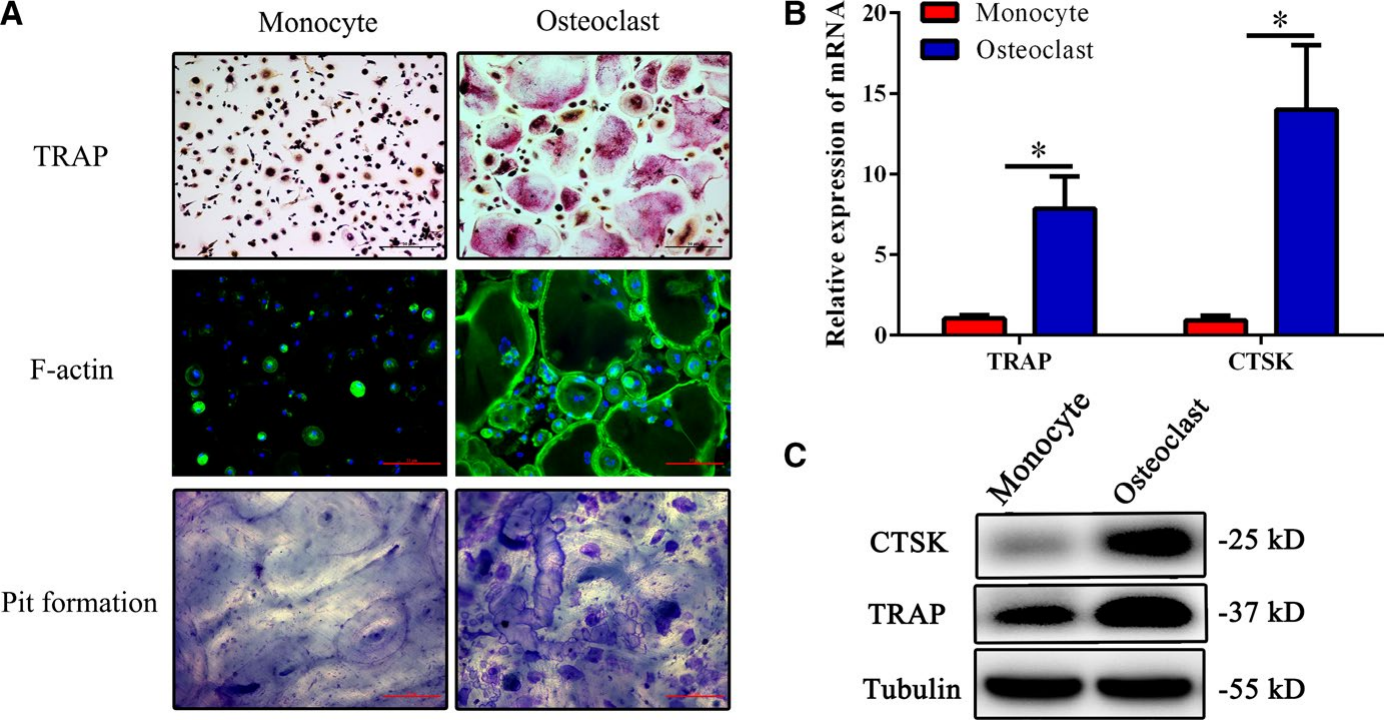
Osteoclast differentiation and identification. Human CD14^+^ monocytes isolated from peripheral blood mononuclear cells (PBMCs) were cultured with RANKL (50 ng/mL) and M‐CSF (25 ng/mL) to induce osteoclasts. A, Representative images of TRAP staining (Scale bar = 40 μm), FITC‐phalloidin staining (Scale bar = 20 μm) and bone resorption assays (Scale bar = 20 μm) in osteoclast differentiation. B, mRNA expression levels of TRAP and CTSK were determined by qPCR. C, Protein levels of TRAP and CTSK were determined by western blot analyses. Values are the mean ± SD of 18 samples per group. The results represent three independent experiments. *P < .05

To induce osteoclast differentiation, we cultured human CD14^+^ monocytes with 50 ng/mL RANKL and 25 ng/mL M‐CSF. TRAP staining was performed on day 9 and TRAP^+^ osteoclast‐like cells were found after culture with M‐CSF and RANKL (Figure 1A). F‐actin assays indicated that these osteoclast‐like cells can cause bone resorption, and this conclusion is further confirmed by pit formation assays (Figure 1A). As shown in Figure 1B,C, osteoclasts expressed higher levels of TRAP and CTSK compared to monocytes, as determined by qRT‐PCR and Western Blot analyses.

### 3.2 Expression profiles of lncRNAs and mRNAs in osteoclast differentiation

**FIGURE 2:**
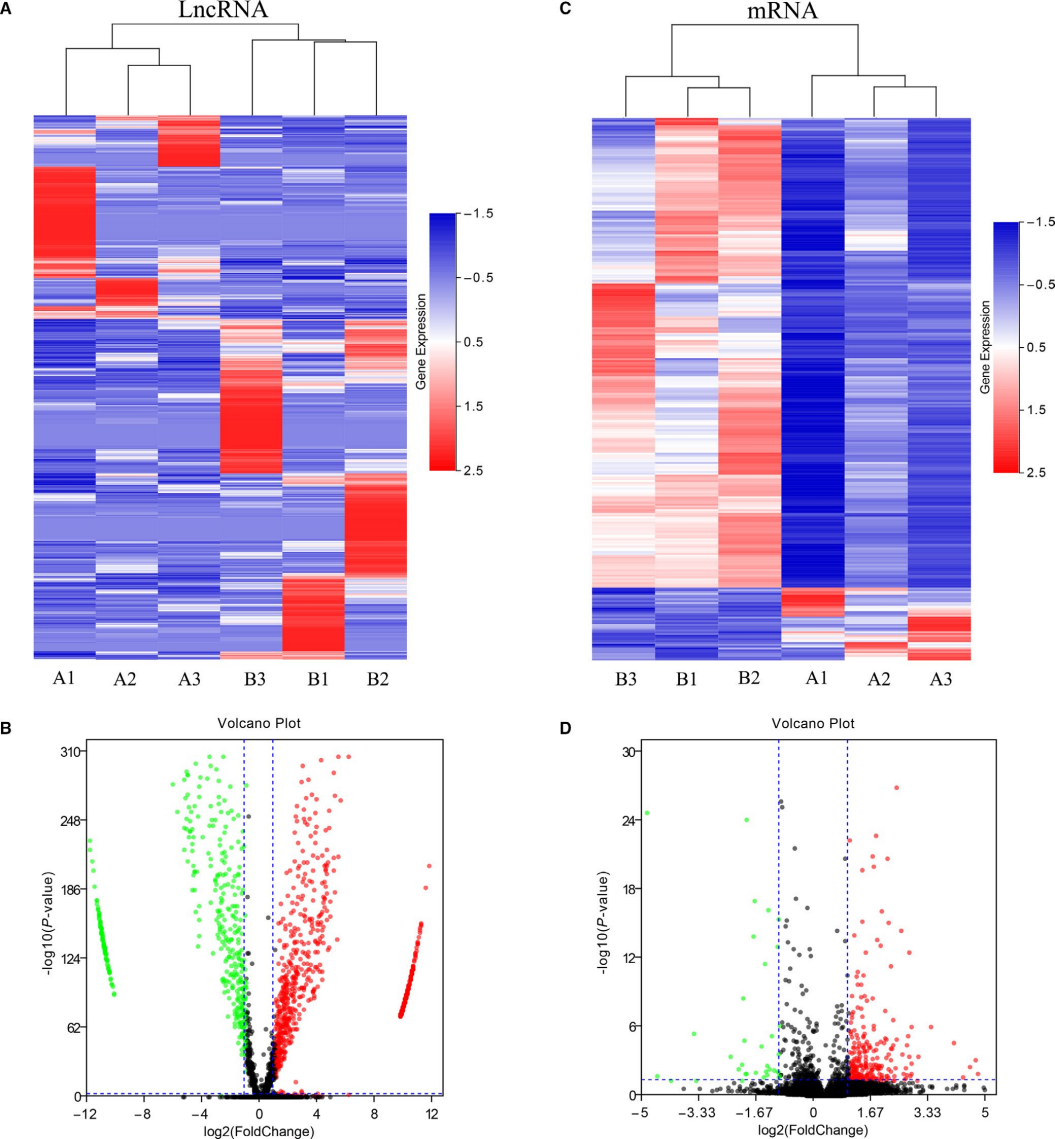
Expression profiles of lncRNAs and mRNAs in osteoclast differentiation. Cluster heat map shows differentially expressed lncRNAs (A) and mRNAs (C) in osteoclast differentiation. The names of the sample groups are on the *x*‐axis and the different profiles are on the *y*‐axis. The red strip indicates high relative expression and the blue strip indicates low relative expression. (A) Control group with monocytes; (B) experimental group with osteoclasts. Volcano plots of lncRNAs (B) and mRNAs (D) in undifferentiated versus differentiated osteoclasts. Red squares in the plots represent the up‐regulated transcripts with statistical significance while green squares represent the down‐regulated transcripts (Fold change ≥ 2.0, *P* < .05)

To identify possible lncRNAs and mRNAs participated in the differentiation of osteoclasts, 17,246 lncRNAs and 11,132 mRNAs expressed in human osteoclasts were analysed by RNA‐seq. As a result, 1117 lncRNAs were differentially expressed in osteoclasts compared to undifferentiated cells, including 699 up‐regulated lncRNAs and 418 down‐regulated lncRNAs, which visualized with a heatmap (Figure 2A) and the volcano plot (Figure 2C). In addition, there were 296 mRNAs differentially expressed in osteoclasts, including 256 up‐regulated mRNAs and 40 down‐regulated mRNAs, as shown in the heatmap (Figure 2B) and the volcano plot (Figure 2D). The 10 lncRNAs and mRNAs with the most significant expression differences are shown in Table 1 and Table 2, respectively.

**TABLE 1:** Top 10 differentially expressed lncRNAs in the expression profile (monocyte vs osteoclast)

Gene symbol	Genome relationship	Fold change	*P*‐value
ENSG00000273301.1	lincRNA	+68.83	0.002992
ENSG00000258580.1	Antisense	+19.02	0.025848
ENSG00000253227.1	lincRNA	+15.25	0.000480
DISC1FP1	lncRNA	+13.26	0.043730
ENSG00000255080.1	lincRNA	+8.20	0.005713
ENSG00000258569.1	lincRNA	+7.22	0.000027
ENSG00000234191.1	lincRNA	+7.07	0.000003
ENSG00000224707.1	Sense_intronic	+6.90	0.032208
ENSG00000257764.2	Antisense	−6.83	0.008987
ENSG00000259225.2	lincRNA	+5.77	0.006423

**TABLE 2:** Top 10 differentially expressed mRNAs in the expression profile (monocyte vs osteoclast)

Gene symbol	Seq name	Fold change	*P*‐value
ENA‐78	NM_002994.4	+60.49	0.000000
PDK4	NM_002612.3	−29.51	0.000000
SULT1B1	NM_014465.3	+27.00	0.012074
S100A3	NM_002960.1	+25.59	0.000711
PALD1	NM_014431.2	−23.72	0.018602
AK5	NM_174858.2	+23.00	0.002836
CKB	NM_001823.4	+21.53	0.000000
EHD2	NM_014601.3	+20.06	0.023756
CLEC10A	NM_182906.3	−18.20	0.040968
PPBP	NM_002704.3	+16.61	0.000023

### 3.3 Validation of the expression profiles by qRT‐PCR

**FIGURE 3:**
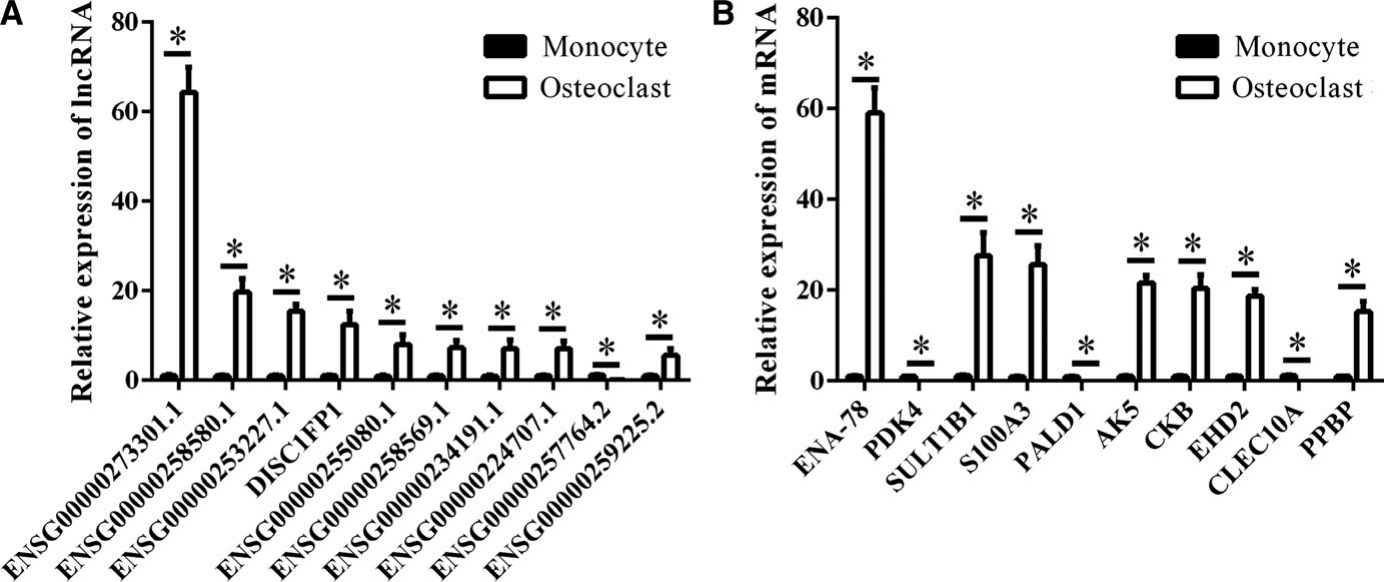
Validation of the expression profiles by qRT‐PCR. Several genes were selected for qRT‐PCR assays. A, The expressions of the top 10 differentially expressed lncRNAs were determined by qRT‐PCR. B, The expressions of the top 10 differentially expressed mRNAs were determined by qRT‐PCR. Data are presented as the mean ± SD (n = 18). The results represent three independent experiments. *P < .05

To confirm the data of RNA‐seq, 10 lncRNAs and mRNAs from above tables were selected for the qRT‐PCR analysis. Their expressions in undifferentiated or differentiated osteoclasts are shown in Figure 3A,B. For lncRNAs, ENSG00000273301.1, ENSG00000258580.1, ENSG00000253227.1, DISC1FP1, ENSG00000255080.1, ENSG00000258569.1, ENSG00000234191.1, ENSG00000224707.1 and ENSG00000259225.2 were up‐regulated, while ENSG00000257764.2 was down‐regulated after differentiation of osteoclasts (Figure 3A). For mRNAs, ENA‐78, SULT1B1, S100A3, AK5, CKB, EHD2 and PPBP were up‐regulated, while PDK4, PALD1 and CLEC10A were down‐regulated after differentiation of osteoclasts (Figure 3B). The results of qRT‐PCR assays almost agreed with the expression profiles detected by RNA‐seq.

### 3.4 GO analysis

**FIGURE 4:**
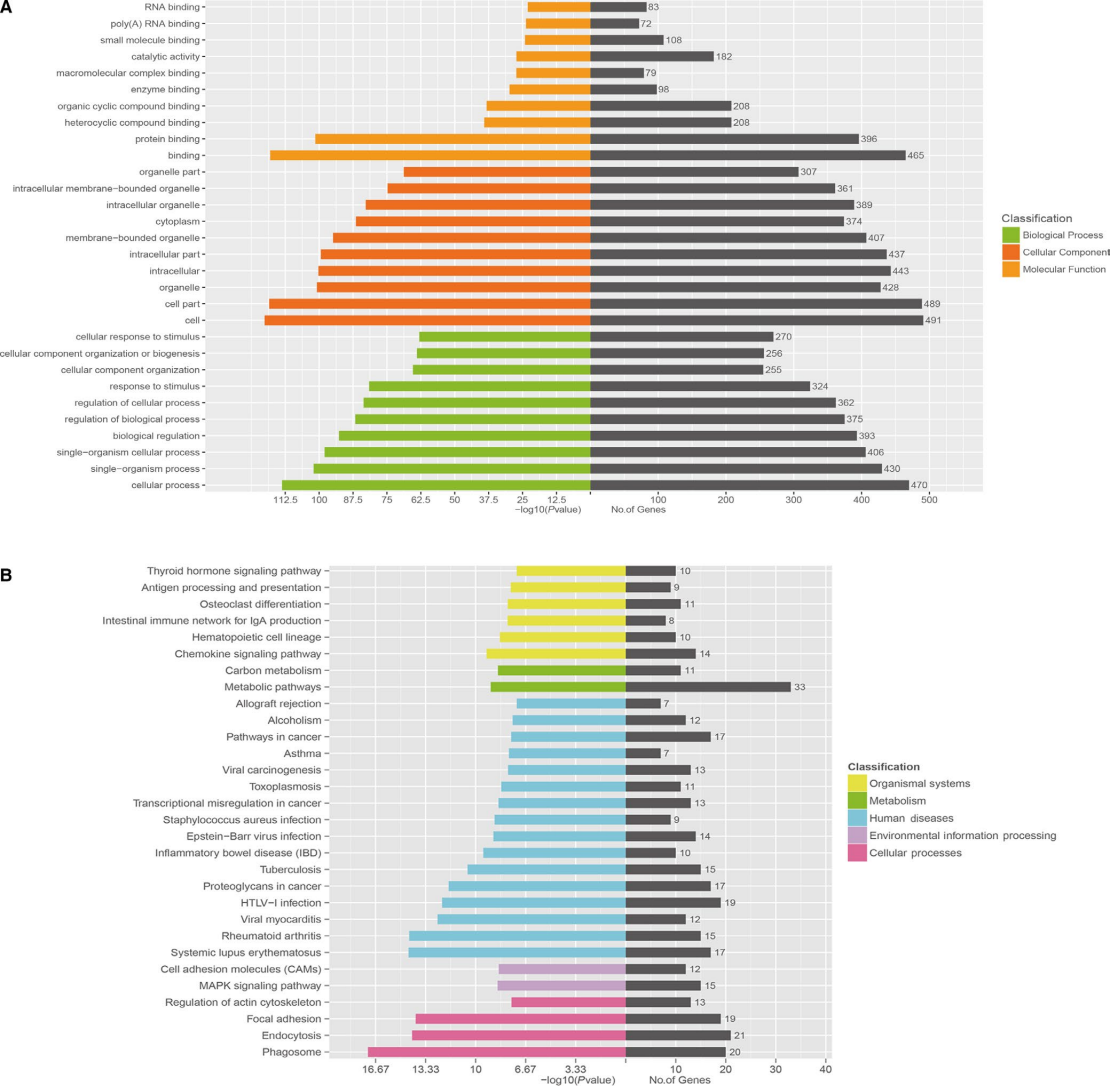
GO analysis and KEGG pathway analysis. A, Top 10 GO terms with significantly differential expression from GO analysis of molecular function, biological process and cellular component are shown. B, Top 30 KEGG pathways with significantly differential expression are shown. Higher ‐LgP values indicate higher significance and lower –LgP values indicate lower significance

GO analysis showed that a total of 2442 GO terms in Biological Process were differentially expressed in osteoclast differentiation, and the three most prevalent GO terms were cellular process, single‐organism process and single‐organism cellular process. A total of 280 GO terms in Cellular Component were differentially expressed in osteoclast differentiation, and the three most prevalent GO terms were cell, cell part and organelle. A total of 374 GO terms in Molecular Function were differentially expressed in osteoclast differentiation, and the three most prevalent GO terms were binding, protein binding and heterocyclic compound binding. The top 10 significantly enriched GO terms are presented in Figure 4A.

### 3.5 Pathway analysis

The KEGG pathway analysis showed that 147 pathways achieved statistically significant enrichment. The most enriched pathways included the MAPK signalling pathway and metabolic pathways (Figure 4B), suggesting that these signalling pathways may play important roles in osteoclast differentiation.

**FIGURE 5:**
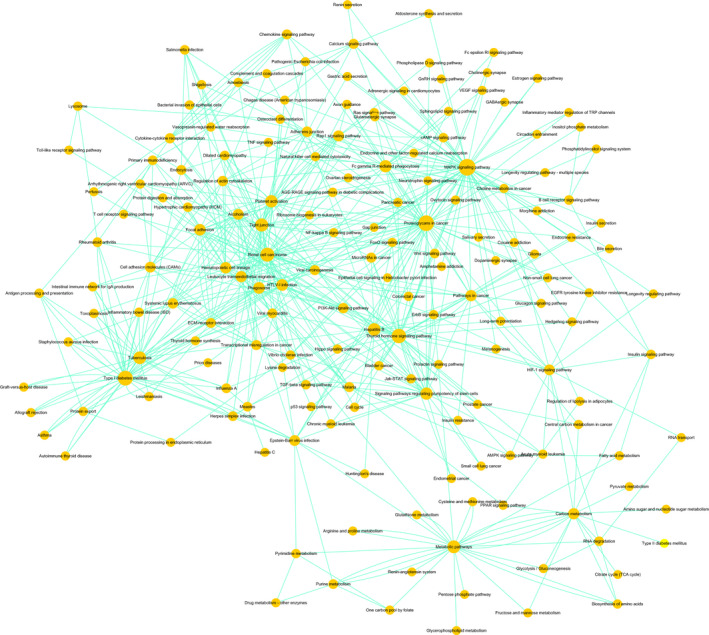
Path‐net analysis. The interaction network for all significantly enriched pathways was done as a Path‐net. Nodes represent different pathways and the size of each circle is determined by the number of other genes that interact with this gene. Mitogen‐activated protein kinase (MAPK) signalling pathway plays a central role with the highest number of interactions with other pathways

The interaction of all significantly enriched pathways was done as a Path‐net (Figure 5). We found that the MAPK signalling pathway obtained the highest number of interactions with other pathways, highlighting its vital role in osteoclast differentiation (Figure 5). Moreover, the PI3K‐AKT signalling pathway and the NF‐ κB signalling pathway also played a significant role in osteoclast differentiation (Figure 5).

To illustrate the correlation of monocytes and mature osteoclasts, gene set enrichment analysis (GSEA) was performed. GSEA plots showed that genes differentially expressed between monocytes and osteoclasts were involved in osteoclast differentiation (Figure S1A). GSEA also revealed that these differentially expressed genes were associated with the MAPK pathway (Figure S1B), supporting the previous results of GO and KEGG analyses.

### 3.6 Construction of the lncRNA‐mRNA co‐expression network

**FIGURE 6:**
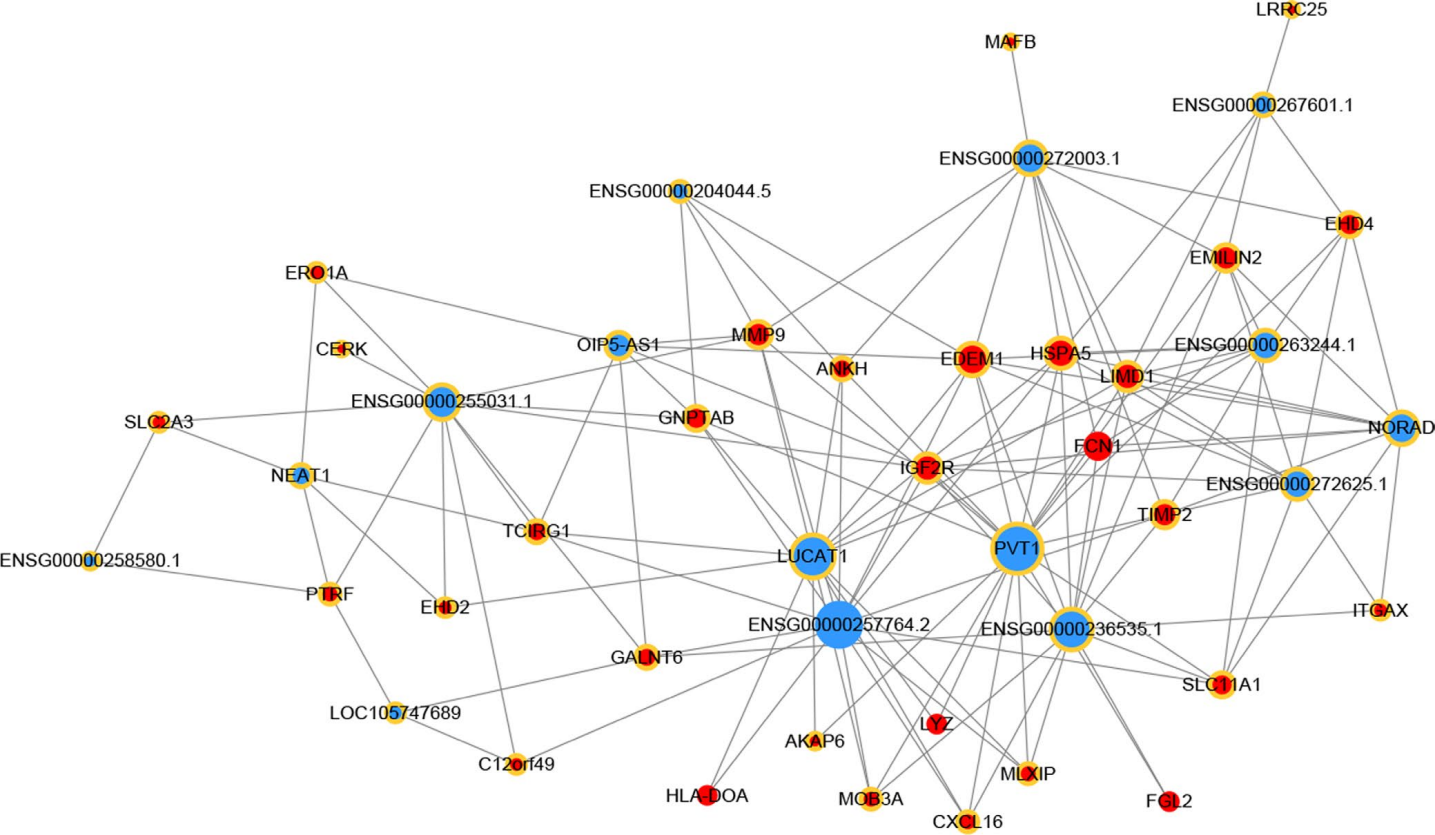
Construction of the lncRNA‐mRNA co‐expression network. A node with a yellow ring indicates lncRNA, and a node without a yellow ring indicates mRNA. Up‐regulated lncRNAs and mRNAs are shown in blue and down‐regulated mRNAs and lncRNAs are shown in red

To explore the potential interactions between mRNAs and lncRNAs, a lncRNA‐mRNA co‐expression network was established. 79 lncRNAs were selected from the differentially expressed lncRNAs and further built as 140 pairs of co‐expression lncRNA‐mRNA (Figure 6). In the co‐expression network, the one that obtained the highest number of interactions was lnRNA ENSG00000257764.2 (Figure 6). Also, we found that ENSG00000257764.2 interacted with TIMP2 (Figure 6).

Top 5 in the lncRNA‐mRNA interaction networks were showed in Table S2. The expressions of lncRNAs and mRNAs were confirmed by qRT‐PCR, and their correlations were analysed by Pearson correlation coefficient. The results showed that lncRNA PVT1 had a strong negative correlation with FCN1, while the other four had a strong positive correlation (Figure S2). These results were almost consistent with the data from the lncRNA‐mRNA interaction networks.

### 3.7 Construction of the competing endogenous RNA network

**FIGURE 7:**
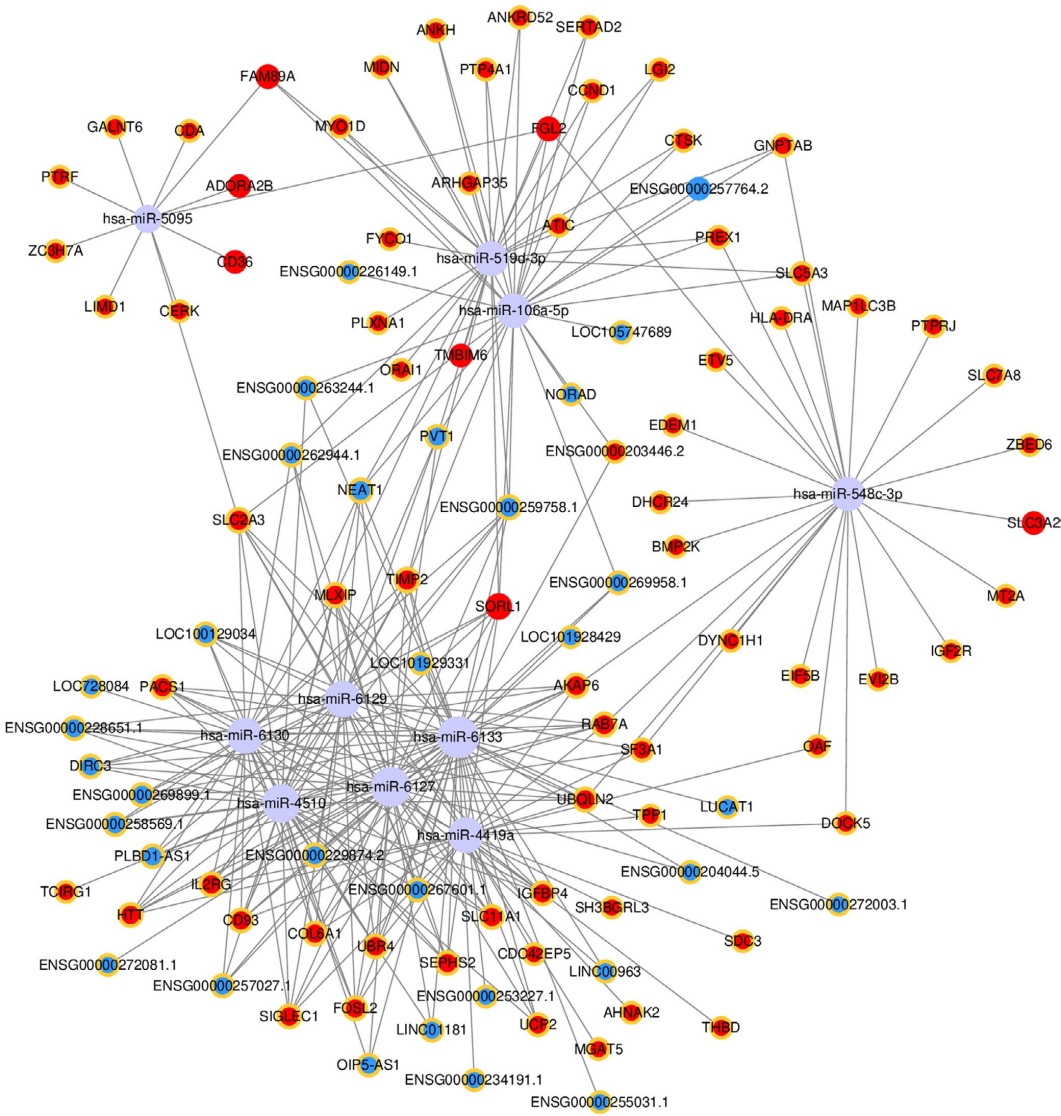
Construction of the competing endogenous RNA network. Nodes with a yellow ring represent up‐regulated RNAs and nodes without a yellow ring represent down‐regulated RNAs. LncRNAs, mRNAs and miRNA are shown in blue, red and grey, respectively. The size of each circle is determined by the number of other genes that interact with this gene

A ceRNA network was constructed to display the interactions between miRNAs, mRNAs and lncRNAs (Figure 7). We found that hsa‐miR‐106a‐5p, hsa‐miR‐548c‐3p and hsa‐miR‐6133 were enriched in this ceRNA network, indicating that these three miRNAs may have important roles in the ceRNA network. Additionally, ENSG00000257764.2 interacted with hsa‐miR‐106a‐5p and hsa‐miR‐106a‐5p combined with TIMP2 in this regulatory network (Figure 7).

## 4 DISCUSSION

In this study, through RNA‐seq analysis, a number of lncRNAs and mRNAs differentially expressed in osteoclast differentiation were identified. Then the cellular events and biological pathways in osteoclast differentiation were identified with GO, KEGG and Path‐net analysis. Furthermore, co‐expression networks presented the interactions between lncRNAs and mRNAs, and the core regulatory factors in osteoclast differentiation. Finally, a ceRNA network was established to display the interactions between lncRNAs and miRNAs. We found that ENSG00000257764.2‐miR‐106a‐5p‐TIMP2 may play a central role in osteoclast differentiation. Our study provides a basis to further understand the role and underlying mechanism of lncRNAs in osteoclast differentiation and many of them could be potential targets for bone metabolic disease.

In recent years, increasing numbers of studies demonstrate that osteoclast differentiation is precisely regulated by molecular signals.^32^, ^33^, ^34^, ^35^, ^36^, ^37^ Besides, lncRNAs play important roles in cell differentiation such as osteogenic differentiation and adipogenic differentiation.^38^, ^39^ However, the role of lncRNAs in osteoclast differentiation remains largely unknown. Emerging evidence revealed the expression profiles of lncRNAs during osteoclast differentiation of RAW264.7 cells.^40^ Some studies showed that lncRNAs were involved in osteoclast differentiation. For example, knockdown of lncRNA DANCR reduced osteoclastogenesis and root resorption induced by compression force via Jagged1.^41^ LncRNA MIRG induced osteoclastogenesis and bone resorption in osteoporosis through negative regulation of miR‐1897.^42^ LncRNA Bmncr alleviates the progression of osteoporosis by inhibiting RANML‐induced osteoclast differentiation.^43^ However, the cells used in these studies were RAW264.7 cells. Since RAW264.7 cells are derived from the Abelson murine leukaemia virus‐induced tumour, it cannot fully represent the biological process of osteoclast differentiation, hence, further studies using primary cells are needed.^40^ Further studies indicated that lncRNAs can regulate osteoclast differentiation.^44^, ^45^ For example, lncRNA LINC00311 promoted osteoclast differentiation in rats by targeting DLL3.^44^ LncRNA AK077216 suppressed the expression of NIP45 and promoted NFATc1 expression, leading to the promotion of osteoclast differentiation.^45^ LncRNA Neat1 stimulated osteoclastogenesis via sponging miR‐7.^46^ LncRNA MALAT1 promoted osteoclast differentiation via sponging miR‐124.^47^ LncRNA Nron inhibited osteoclast differentiation by reducing the nuclear import of Nfatc1.^48^ Although these lncRNA are reported to regulate osteoclast differentiation, the role of other lncRNAs is still needed to be further explored. Moreover, these studies used cells from mice. Our study used human cells and so, in comparison with other studies, the outcomes may be more suitable for research of human diseases.

In our study, the altered lncRNAs and mRNAs in osteoclast differentiation were identified using RNA‐seq technology. The qRT‐PCR results agreed with the RNA‐seq data and confirmed the accuracy of the RNA‐seq analysis.

Then we performed GO and KEGG pathway analyses to predict the functions of the differentially expressed lncRNAs and mRNAs. The GO analysis found significant enrichments in thousands of GO terms and the KEGG pathway analysis identified the important pathways involved in osteoclast differentiation. We further constructed a Path‐net to investigate the interaction of these significant pathways. The results of Path‐net analysis revealed the importance of the NF‐κB pathway in osteoclast differentiation. In fact, previous studies have shown that the NF‐κB pathway is the canonical pathway of osteoclast differentiation.^49^, ^50^, ^51^, ^52^ This demonstrates that Path‐net analysis is an accurate and effective way to find the significant pathways involved in osteoclast differentiation. Recently, some researchers have found that non‐canonical pathways also take part in osteoclast differentiation.^53^ He et al showed that the p38 pathway in myeloma cells regulated osteoclast differentiation.^54^ In fact, p38, together with ERK and JNK, belong to the MAPK signalling pathway.^55^ In our study, as shown in Figure 5, we found that the MAPK signalling pathway played the central role in osteoclast differentiation since it acquired the highest number of interactions with other pathways. Further studies are needed to explore its specific function in regulating osteoclast differentiation.

Regarding the regulatory functions of lncRNAs, we constructed the lncRNA‐mRNA co‐expression network and found that lncRNA ENSG00000257764.2 obtained the highest number of interactions and interacted with TIMP2. TIMP2 are inhibitors of matrix metalloproteinases (MMPs), which are a group of endopeptidases that regulate the formation and function of osteoclast.^56^ In the qRT‐PCR results mentioned above, we found that ENSG00000257764.2 was significantly down‐regulated in the differentiation of osteoclast. These results indicated that ENSG00000257764.2 may play a central role in regulating osteoclast differentiation by regulating the expression of TIMP2. However, its significant roles have not been studied in osteoclast differentiation and further experiments are required to test this hypothesis.

LncRNAs carry out their functions through a variety of mechanisms including the ceRNA hypothesis.^29^, ^57^ The ceRNA hypothesis suggests that transcripts with common miRNA binding sites compete for the post‐transcriptional regulation of genes.^29^, ^57^ This hypothesis has attracted extensive attention these years as an important functional mechanism of lncRNAs. However, there are no studies that link miRNA and lncRNA in osteoclast differentiation. In our study, a ceRNA network was established for the differentially expressed lncRNAs. We found that hsa‐miR‐106a‐5p was enriched in the ceRNA network, and interacted not only with ENSG00000257764.2, but also TIMP2. Therefore, we hypothesized that ENSG00000257764.2‐miR‐106a‐5p‐TIMP2 may be a crucial axis in the regulation network of osteoclast differentiation since it interacts with the greatest number of differentially expressed mRNAs and lncRNAs. However, the role of ENSG00000257764.2‐miR‐106a‐5p‐TIMP2 in osteoclast differentiation remains unknown and further studies are necessary.

In conclusion, we investigated the lncRNA‐mRNA expression profiles in osteoclast differentiation through RNA‐seq and identified the potential regulatory mechanisms via bioinformatic analyses. We aim to reveal the role of lncRNAs in osteoclast differentiation and osteoclast‐related bone disorders. Our results may help illuminate the mechanism of osteoclast differentiation and provide potential targets for osteoclast‐related bone disorders. However, our study also has some limitations, such as a lack of functional research of these differentially expressed lncRNAs. More studies are needed to explore the roles of these differentially expressed lncRNAs in osteoclast differentiation.

## CONFLICT OF INTEREST

The authors confirm that there are no conflicts of interest.

## AUTHOR CONTRIBUTION

**Wenjie Liu:** Conceptualization (equal); Data curation (equal); Formal analysis (equal); Writing‐original draft (equal). **Zhaofeng Li:** Formal analysis (equal); Investigation (equal). **Zhaopeng Cai:** Formal analysis (equal); Investigation (equal). **Zhongyu Xie:** Methodology (equal). **Jinteng Li:** Supervision (equal). **Ming Li:** Software (equal). **Shuizhong Cen:** Validation (equal). **Su'an Tang:** Validation (equal). **Guan Zheng:** Visualization (equal). **Guiwen Ye:** Software (equal). **Hongjun Su:** Resources (equal). **Shan Wang:** Resources (equal). **Peng Wang:** Funding acquisition (equal); Supervision (equal); Writing‐review & editing (equal). **Huiyong Shen:** Funding acquisition (equal); Supervision (equal); Writing‐review & editing (equal). **Yanfeng Wu:** Funding acquisition (equal); Project administration (equal); Resources (equal); Supervision (equal); Writing‐review & editing (equal).

## Supporting information

Appendix S1

## Data Availability

The data that support the findings of this study are available from the corresponding author on request.
