# Supplementary material for: LncRNA‐mRNA expression profiles and functional networks in osteoclast differentiation
Source: J Cell Mol Med. 2020 Jul 26;24(17):9786–97. doi: 10.1111/jcmm.15560 (PMC7520269; doi:10.1111/jcmm.15560)
Supplement: Supplementary file 1 — Appendix S1 [file JCMM-24-9786-s001.docx]

**Supplementary Tables**

**Supplementary Table S1 Primer sequences used for qRT-PCR**

| Gene Name | Forward Primer | Reverse Primer |
| --- | --- | --- |
| GAPDH | AAGGTGAAGGTCGGAGTCAA | AATGAAGGGGTCATTGATGG |
| TRAP | TGAGGACGTATTCTCTGACCG | CACATTGGTCTGTGGGATCTTG |
| CTSK | GCAGAAGAACCGGGGTATTGA | GAAGGAGGTCAGGCTTGCAT |
| ENSG00000273301.1 | TTGGAGGAGACCAGGGTTCA | GCCACTGGTCCCACTTTCTT |
| ENSG00000258580.1 | GCTATGGTCCCTCTCCCAAG | ATGCACATCAAGGCTTCTGGA |
| ENSG00000253227.1 | CCTTAGGGGTGTGGACACTG | GCCTTGACAGCTTAGTGGCT |
| DISC1FP1 | GAAGAAGCTACCTGGGCCTG | AGCATTGAAACACGGAGGCT |
| ENSG00000255080.1 | CAGGCTACTGCTGGGACATT | TCTGTCCCATGTGCTTCACG |
| ENSG00000258569.1 | GTCCCAAGTTACCCCAAGGA | CACGTTGAAAACGTCCTCCG |
| ENSG00000234191.1 | AATAGAGCCTGCCAGAAGGG | CACAGGTTGAGCAGGTCCAT |
| ENSG00000224707.1 | AGCGGCGCTTGGTAATAAGA | CGCCATTGCTTTCTCCATCG |
| ENSG00000257764.2 | TCTCAATTCAGGCAGTCTGTT | CAAAATACCAGCTGATGAAGGCA |
| ENSG00000259225.2 | CCTGCATGCACAAAAGGGTC | CGGGCTGATGACGATCTGAA |
| ENA-78 | TGTGCAATTAACAAAGCTACTGC | AGGCATCTAAAAAGCTCAGCA |
| PDK4 | GGAGCATTTCTCGCGCTACA | ACAGGCAATTCTTGTCGCAAA |
| SULT1B1 | TTGAACAGTTCCATAGCAGACC | CAGGGAGAGTCATTTCCAACATT |
| S100A3 | GTGGGGACAAATACAAGCTCTG | GTCACATTCCCGAAACTCAGT |
| PALD1 | CAGTGGCACGATGGACAGTC | CCCAACGTGTAATGAGCCTTG |
| AK5 | TCTAAGCCCGAAGATCCAGTAG | GTGACTGTCCTCCATTTAGTGG |
| CKB | GCTGCGACTTCAGAAGCGA | GGCATGAGGTCGTCGATGG |
| EHD2 | TCCGCAAACTCAACCCTTTC | TCTCCAGGACCTGATTAGGGA |
| CLEC10A | AGCAACTTCACCTCAAACACTG | AGATGCTATCGTTTCTTCCAAGC |
| PPBP | GTAACAGTGCGAGACCACTTC | CTTTGCCTTTCGCCAAGTTTC |

**Supplementary Table S2 Top 5 in the lncRNA-mRNA interaction networks**

|  | LncRNA | mRNA | Correlation coefficient |
| --- | --- | --- | --- |
| 1 | PVT1 | FCN1 | -0. 992 |
| 2 | PVT1 | LIMD1 | 0.986 |
| 3 | ENSG00000236535.1 | TIMP2 | 0.983 |
| 4 | ENSG00000255031.1 | CERK | 0.981 |
| 5 | PVT1 | HSPA5 | 0.980 |

**Supplementary Figures**

**Supplementary Figure S1:**


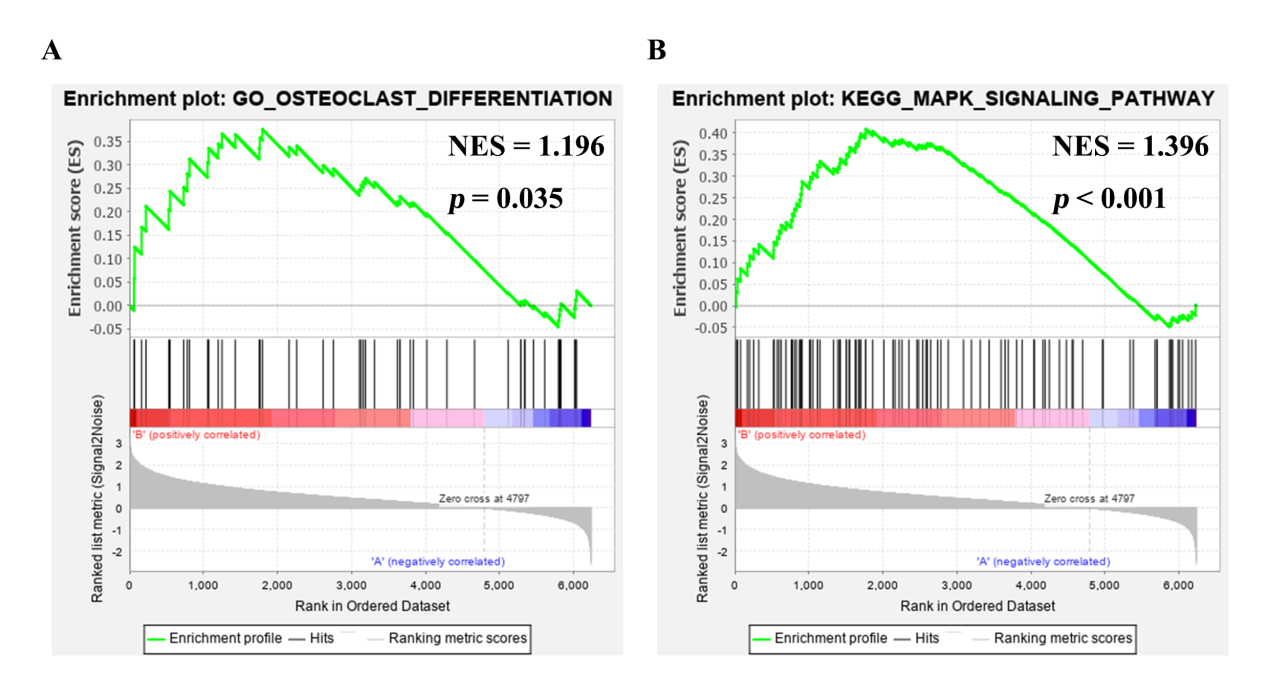


**Figure S1 Gene set enrichment analysis.** (A) GSEA plots showed that genes differentially expressed between monocytes and osteoclasts were involved in osteoclast differentiation. (B) GSEA plots showed that the differentially expressed genes in the RNA-seq analysis were associated with the MAPK pathway.

**Supplementary Figure S2:**


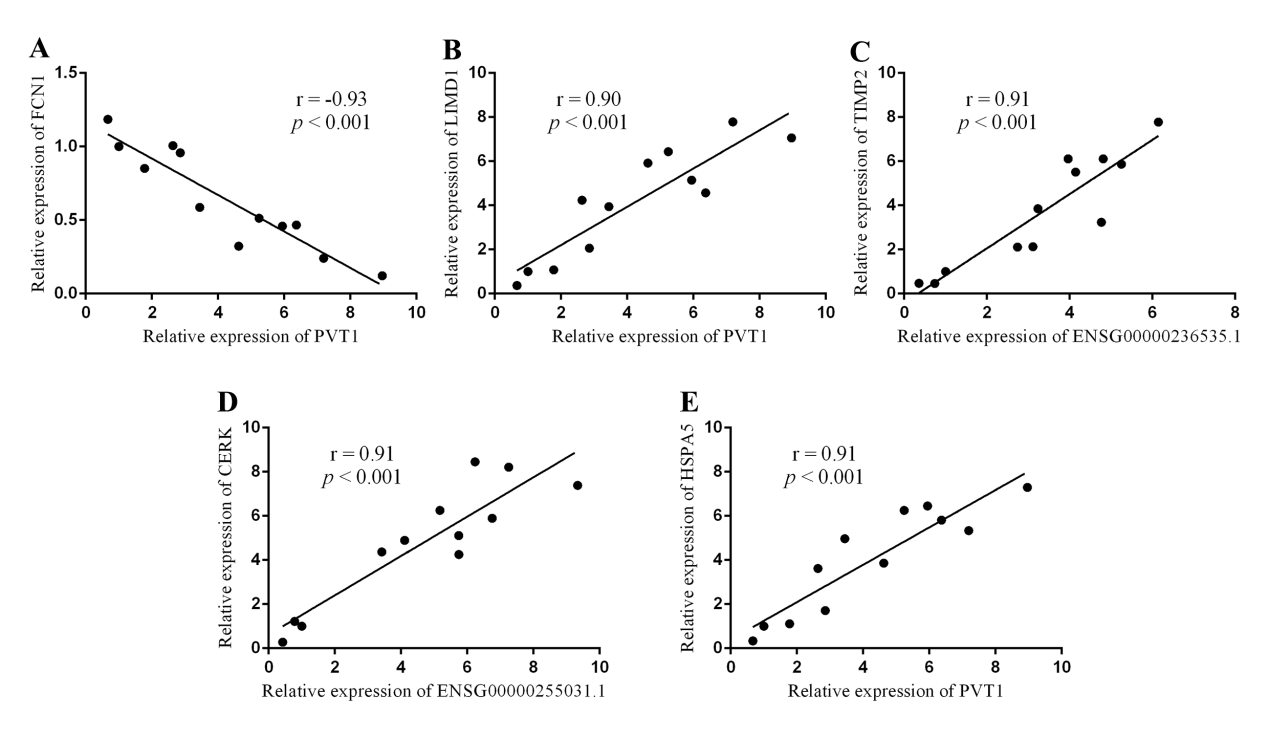


**Figure S2 The correlation of lncRNAs and mRNAs.** (A) The lncRNA PVT1 negatively correlates with FCN1. (B) The lncRNA PVT1 positively correlates with LIMD1. (C) The lncRNA ENSG00000236535.1 positively correlates with TIMP2. (D) The lncRNA ENSG00000255031.1 positively correlates with LIMD1. (E) The lncRNA PVT1 positively correlates with HSPA5. The correlation was determined by Pearson Correlation Coefficient. n = 12.
